# Comparison of characteristics and outcomes on ETP-ALL/LBL and non-ETP ALL patients receiving allogeneic hematopoietic stem cell transplantation

**DOI:** 10.3389/fonc.2022.1025885

**Published:** 2023-01-04

**Authors:** Juan Chen, Li Liu, Runzhi Ma, Aiming Pang, Donglin Yang, Xin Chen, Jialin Wei, Yi He, Rongli Zhang, Weihua Zhai, Qiaoling Ma, Erlie Jiang, Mingzhe Han, Sizhou Feng

**Affiliations:** ^1^ State Key Laboratory of Experimental Hematology, National Clinical Research Center for Blood Diseases, Haihe Laboratory of Cell Ecosystem, Institute of Hematology and Blood Diseases Hospital, Chinese Academy of Medical Sciences and Peking Union Medical College, Tianjin, China; ^2^ Department of Hematology, The First Affiliated Hospital of Soochow University, National Clinical Research Center for Hematologic Diseases, Soochow University, Suzhou, China; ^3^ Key Laboratory of Thrombosis and Hemostasis of Ministry of Health, Jiangsu Institute of Hematology, The First Affiliated Hospital of Soochow University, Collaborative Innovation Center of Hematology, Suzhou, China

**Keywords:** early T-cell precursors, acute lymphoblastic leukemia, allogeneic hematopoietic stem cell transplantation, characteristics, prognosis

## Abstract

**Objective:**

This study aims to compare the characteristics of early T-cell precursor acute lymphoblastic leukemia/lymphoma (ETP-ALL/LBL) and non-ETP ALL patients and the outcomes of these patients after allogeneic hematopoietic stem cell transplantation (allo-HSCT).

**Method:**

A total of 57 patients with T-cell acute lymphoblastic leukemia/lymphoma receiving allo-HSCT at our center between January 2016 and March 2022 were enrolled in the study. Twenty-eight patients were diagnosed as ETP-ALL/LBL (28/57, 49.12%) in the cohort.

**Results:**

The baseline characteristic was not significantly different between the two groups. The median time for myeloid engraftment was 14 days (ranged from 11 to 21) *versus* 14 days (ranged from 10 to 20) (*P* = 0.067) and 18 days (ranged from 12 to 27) *versus* 15.5 days (ranged from 12 to 72) (*P* = 0.183) for platelet engraftment in the ETP-ALL/LBL and non-ETP ALL groups, respectively. There was no significant difference in 5-year overall survival (54.74% ± 10.33% *vs*. 64.20% ± 10.30%, *P* = 0.786), relapse-free survival (56.22% ± 10.11% *vs*. 57.17% ± 12.71%, *P* = 0.841), cumulative incidence of relapse (30.14% ± 9.85% *vs*. 22.79% ± 8.24%, *P* = 0.774), and non-relapse mortality (19.52% ± 8.99% *vs*. 25.95% ± 14.44%, *P* = 0.967) between the two groups. The incidence of acute graft *versus* host disease (aGVHD) (*P* = 0.922), II–IV aGVHD (*P* = 0.940), III–IV aGVHD (*P* = 0.664), cytomegalovirus infection (*P* = 0.862), Epstein–Barr virus infection (*P* = 0.610), and severe bacterial infection (*P* = 0.145) was also similar.

**Conclusion:**

The prognosis of patients with ETP-ALL/LBL was similar to non-ETP ALL patients when they received allo-HSCT.

## Introduction

T-cell acute lymphoblastic leukemia (T-ALL) is an aggressive hematological malignancy which accounts for 15% and 25% of childhood and adult ALL cases, respectively (1). Early T-cell precursor lymphoblastic leukemia/lymphoma (ETP-ALL/LBL) is a special subtype of T-ALL first recognized in 2009 (2), which is characterized by arrested early T-cell differentiation, with some myeloid and stem cell characteristics remaining at the immunophenotypic and also genetic levels (3, 4). The incidence of ETP-ALL reported in previous studies is 11%–16% of T-ALL cases in children and 7.4%–32% in adults (5–7), respectively. In a large cohort study in Chinese adult T-ALL (*n* = 112), ETP-ALL accounts for 47.3% of all patients (8). Some studies suggested that the prognosis of ETP-ALL/LBL was worse than that of typical T-ALL (2, 5, 9–11). However, other studies have found that the prognosis of ETP-ALL and non-ETP was not significantly different (8, 12–14).

Although many efforts have been made to uncover the genetic aberrations and molecular pathogenesis of ETP-ALL (15–18), the management of ETP-ALL is still challenging. Allogeneic hematopoietic stem cell transplantation (allo-HSCT) is an important potentially curative treatment for ETP-ALL/LBL. In this study, we aim to assess the efficacy of allo-HSCT on ETP-ALL/LBL patients and compare the outcomes of ETP-ALL/LBL and non-ETP patients.

## Methods

### Patients and definitions

We retrospectively analyzed the data of 57 patients who received allo-HSCT in our center from January 2016 to March 2022. The final date of follow-up was June 30, 2022 for patients without events. Of the 57 patients, 28 were diagnosed as ETP-ALL/LBL (one patient was diagnosed as ETP-LBL) according to the diagnosis criteria. ETP was diagnosed by the immunophenotype of the positive expression of CD7 but lack of CD1a and CD8, weak expression of CD5 (with <75% positive blasts), and positive expression of one or more stem cell or myeloid markers including CD117, HLA-DR, CD13, CD33, CD11b, or CD65 (3). The initial induction chemotherapy was VDCLP or Hyper-CVAD. After complete remission, we conducted three to six courses of consolidation chemotherapy before allo-HSCT. Minimal residual disease (MRD) analysis was detected by flow cytometry, and MRD <0.01% (1 * 10^-4^) of nucleated cells was defined as negative. All patients and donors provided written informed consent for this protocol. For patients younger than 18 years old in the cohort, the consent was carried out by their parents. This study was approved by the Ethics Review Committee of the Institute of Hematology, Chinese Academy of Medical Science and Peking Union Medical College, and was in compliance with the Declaration of Helsinki.

### Treatment

All the patients received a myeloablative conditioning regimen before allo-HSCT, including total body irradiation/Cy-based regime [(3.33 Gy, -9 to -7 days) + Cy (cyclophosphamide) (40 mg/kg/day, -6 to -5 days) + Ara-c (cytarabine) (2 g/m^2^/day, -4 to -2 days) + Flu (fludarabine) (30 mg/m^2^/day, -4 to -2 days)] and Bu/Cy-based regime [Bu (busulfan) (3.2 mg/kg/day, -6 to -4 days) + CTX (cyclophosphamide) (40 mg/kg/day, -6 to -5 days) + VP-16 (etoposide) (20 mg/kg/day, -9 to -7 days). For patients who received grafts from HLA-haploidentical related donor and unrelated donor, additional anti-thymocyte globulin/anti-lymphocyte globulin (anti-thymocyte globulin 2.5 mg/kg/day, -5 to -2 days/anti-lymphocyte 20 mg/kg/day, -4 to -2 days) was added in the conditioning regimen.

Graft *versus* host disease (GVHD) prophylaxis and supportive care were as described previously (19).

### Criteria of outcomes

Engraftment was defined as absolute neutrophil counts (ANC) ≥0.5 × 10^9^/L for 3 consecutive days and platelet count ≥20 × 10^9^/L without transfusion for seven consecutive days. The Mount Sinai Acute GVHD International Consortium criteria were used to diagnose and grade acute GVHD (aGVHD) (20). Cytomegalovirus (CMV) and Epstein–Barr virus (EBV) viremia was defined as before (19). Severe bacterial infection referred to bacteremia or severe tissue infections. Complete remission (CR) referred to no blasts in blood, ANC >1.0 × 10^9^/L, platelets >100 × 10^9^/L, <5% bone marrow blasts, and no extramedullary leukemia. Overall survival (OS) was calculated from HSCT to death of any cause or last follow-up. Relapse-free survival (RFS) was defined as the time from HSCT to relapse, censoring at death or last follow-up. Cumulative incidence of relapse (CIR) was defined as relapse after HSCT.

### Statistical analysis

The data were analyzed by the software GraphPad Prism 8 (version 8, supplied by GraphPad Software, Inc.) and IBM SPSS statistics 25 (version 25, supplied by IBM). The descriptive statistics for continuous variables and chi-square test and Fisher’s exact test for categorical variables were used to compare incidence in univariate analysis. The Kaplan–Meier method was used to estimate the cumulative survival/incidence, and differences were compared by the log-rank/Wilcoxon test. A two-sided *P*-value <0.05 was considered as statistically significant.

## Results

### Characteristics of patients

**Figure 1:**
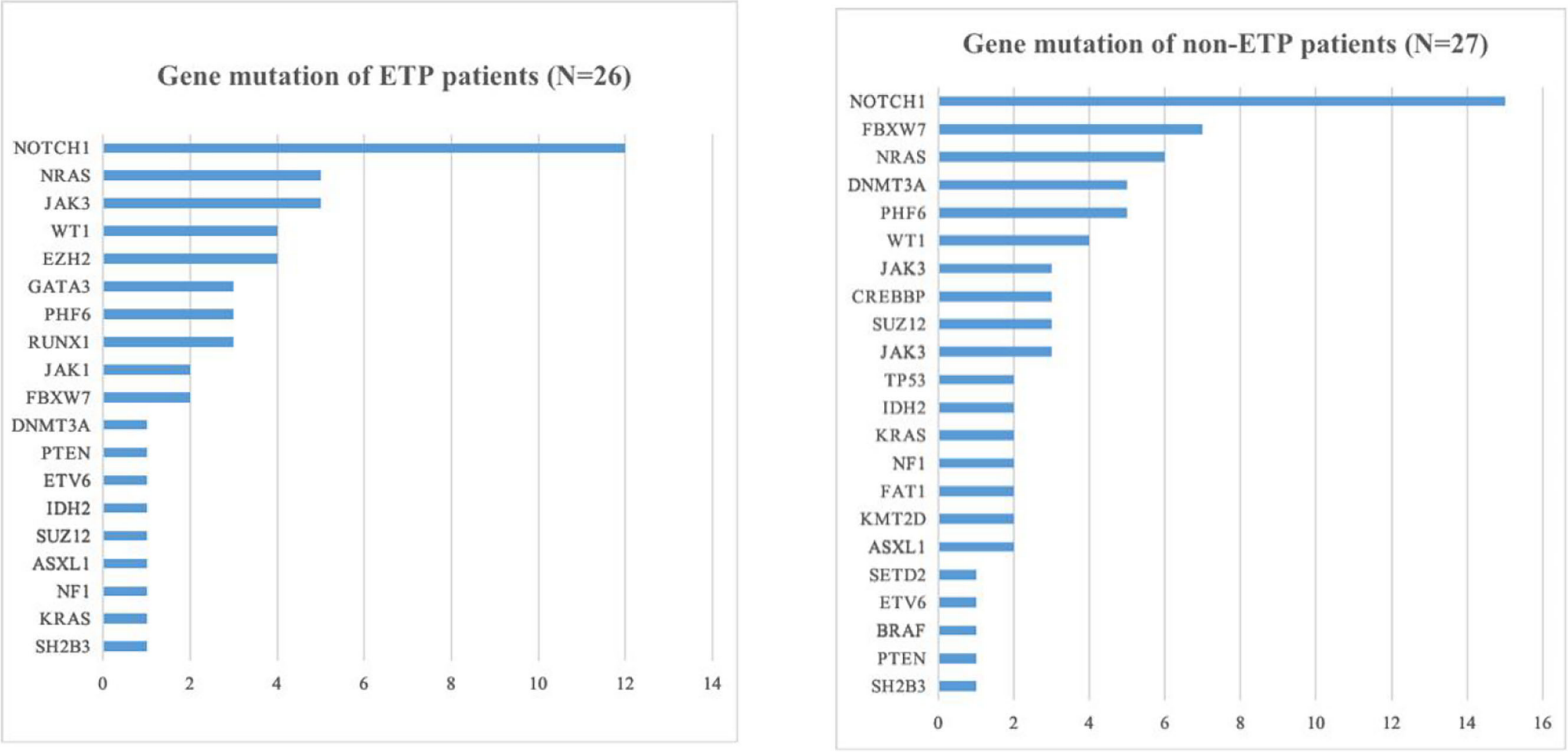
Gene mutation of early T-cell precursor (ETP) and non-ETP patients.

There are 28 and 29 patients in the ETP group and non-ETP group, respectively. The baseline characteristics of patients in the two groups are listed in **Table 1**. Gender, age, WBC/HB/PLT at diagnosis, BM blast, chromosome karyotype, and interval from diagnosis to HSCT did not differ among the two groups. There are 26 and 27 patients in the ETP group and non-ETP group who underwent next-generation sequencing. The top mutated gene in both groups was NOTCH1 (12/26, 46.2% in the ETP group and 15/27, 55.6% in the non-ETP group), followed by NRAS, JAK3, WT1, EZH2 in the ETP group and FBXW7, NRAS, DNMT3A, and PHF6 in the non-ETP group (**Figure 1**).

**Table 1:** Characteristics of patients.

Characteristics	ETP (*N* = 28)	Non-ETP (*N* = 29)	*P*-value
Gender			
Male	24 (85.7%)	19 (65.5%)	0.077
Female	4 (14.3%)	10 (34.5%)	
Age (years)			
Median (range)	26 (16–48)	22 (11–56)	0.570
WBC (×10^9^/L) at diagnosis			
Median (range)	26.00 (1.48–305.64)	51.93 (2.14–461.63)	0.158
HB (g/L) at diagnosis			
Median (range)	105.5 (53.0–165.0)	114.5 (64.2–161.0)	0.157
PLT (×10^9^/L) at diagnosis			
Median (range)	80.0 (25.0–327.0)	53.5 (10.0–270.0)	0.173
BM blast (%)			
Median (range)	87.00 (6.02–96.31)	78.20 (40.00–93.61)	0.866
Chromosome karyotype			
Normal	14 (50.0%)	19 (65.5%)	0.308
Abnormal	11 (39. 3%)	6 (20.7%)	
Unknown	3 (10.7%)	4 (13.8%)	
Interval from diagnosis to hematopoietic stem cell transplantation (days)			
Median (range)	218 (116–380)	209 (48–352)	0.297

ETP, early T-cell precursor; WBC, white blood cell; HB, hemoglobin; PLT, platelet; BM, bone marrow.

### Transplantation details

The transplantation-associated details including donor type, chemotherapy before CR, MRD status before transplantation, GVHD prophylaxis, and dose of MNC and CD34^+^ cells between the two groups were similar (**Table 2**). The median dose of infused MNC and CD34^+^ cells in the ETP group was 10.83 × 10^8^/kg (range, 6.76–21.10) and 3.05 × 10^6^/kg (range, 1.56–5.90), which was not significantly different from the non-ETP group [MNC: 11.79 × 10^8^/kg (range, 7.00–23.84) and CD34^+^ cells: 3.00 × 10^6^/kg (range, 2.00–9.48)]. Moreover, there was one patient in each group who received additional cord blood infusion due to insufficient infused cell dose.

**Table 2:** Transplantation details.

	ETP (*N* = 28)	Non-ETP (*N* = 29)	*P*-value
Donor type			
MSD	10 (35.7%)	11 (37.9%)	0.104
HRD	14 (50.0%)	18 (62.1%)	
MUD	4 (14.3%)	0 (0.0%)	
Cycles of chemotherapy before CR			
1	18 (64.3%)	23 (79.3%)	0.207
≥2	10 (35.7%)	6 (20.7%)	
MRD status before transplantation			
Positive	7 (25.0%)	6 (20.7%)	0.698
Negative	21 (75.0%)	23 (79.3%)	
GVHD prophylaxis			
CSA	9 (32.1%)	13 (44.8%)	0.325
FK506	19 (67.9%)	16 (55.2%)	
Dose of MNC (*10^8^/kg)			
Median (range)	10.83 (6.76–21.10)	11.79 (7.00–23.84)	0.260
Dose of CD34+ cells (*10^6^/kg)			
Median (range)	3.05 (1.56–5.90)	3.00 (2.00–9.48)	0.503

ETP, early T-cell precursor; MSD, matched sibling donor; HRD, HLA-haploidentical related donor; MUD, matched unrelated donor; CR complete remission; MRD, minimal residual disease; GVHD, graft versus host disease; MNC, mononuclear cell.

### Engraftment

All patients had ANC engraftment, whereas 25 patients (89.3%) in the ETP group and 28 patients (96.6%) in the non-ETP group had platelet engraftment in 100 days post-transplantation. The median time of ANC recovery in the ETP group and non-ETP group was 14 days (ranged from 11 to 21) and 14 days (ranged from 10 to 20), respectively. For platelet recovery, the median time was 18 days (ranged from 12 to 27) and 15.5 days (ranged from 12 to 72), respectively.

### Infection and GVHD

The incidence of CMV viremia and EBV viremia was not significantly different in the ETP group and the non-ETP group (64.3% *vs*. 62.1%, *P* = 0.862; 10.7% *vs*. 6.9%, *P* = 0.610, respectively). In total, 14 patients in the ETP group and nine patients in the non-ETP group developed severe infection (50.0% *vs*. 31.0%, *P* = 0.431). The incidence of I–IV, II–IV, and III–IV aGVHD was similar in the two groups (*P* = 0.922; *P* = 0.940; *P* = 0.664).

### Deaths and survival

**Figure 2:**
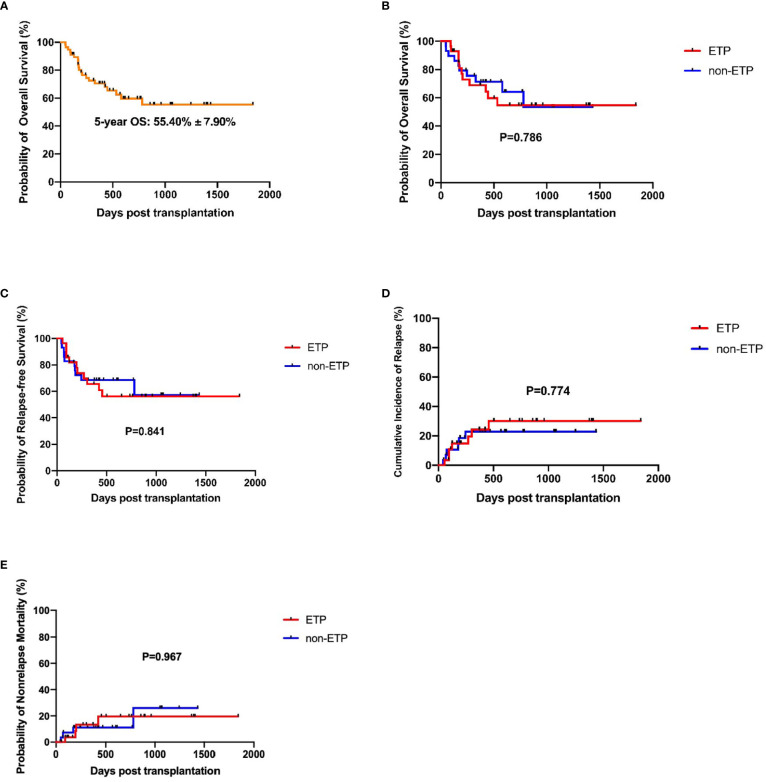
Survival analysis of early T-cell precursor (ETP) and non-ETP patients. **(A)** Overall survival of the total cohort; **(B)** Overall survival of ETP and non-ETP patients; **(C)** Relapse-free survival of ETP and non-ETP patients; **(D)** Cumulative incidence of relapse of ETP and non-ETP patients; **(E)** Non-relapse mortality of ETP and non-ETP patients.

**Figure 3:**
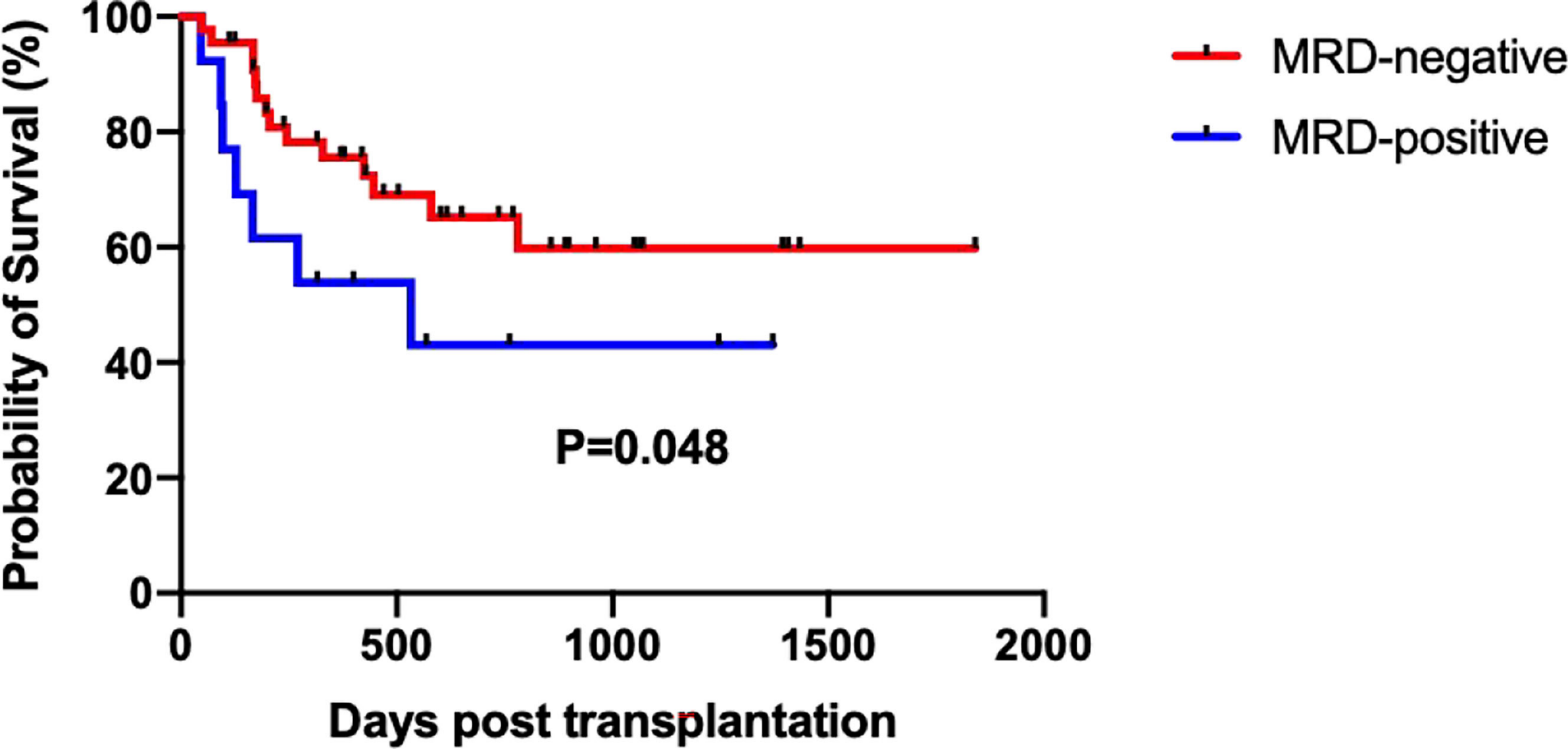
Survival of minimal residual disease (MRD)-positive and MRD-negative patients.

The median time from HSCT to death or last follow-up was 424 days (ranged from 46 to 1841). The estimated 5-year OS of the total cohort was 55.40% ± 7.90% (**Figure 2A**). Until the last follow-up, there were 11 patients who died in the ETP group, seven had a relapse, two had infection or aGVHD, and one had graft failure. A total of 10 patients died in the non-ETP group, six had a relapse and four had infection or aGVHD. In the ETP group, at a median follow-up of 435 days (ranges from 93 to 1,841), 17 patients survived, and the 5-year OS was 54.74% ± 10.33%. In the non-ETP group, at a median follow-up of 419 days (ranged from 46 to 1,434), 19 patients survived, and the 5-year OS was 64.20% ± 10.30%. There was no significant difference in terms of the 5-year OS between the two groups (*P* = 0.786), and so were the 5-year RFS, CIR, and non-relapse mortality (NRM) (*P* = 0.841; *P* = 0.774; *P* = 0.697) (**Table 3** and **Figures 2B–E**). Moreover, we compared the survival of MRD-positive and MRD-negative patients. Patients who were MRD-negative before transplantation had a higher 5-year OS than the MRD-positive patients (59.79% ± 9.04% *vs*. 43.08% ± 14.67%, *P* = 0.048) (**Figure 3**).

**Table 3:** Outcomes of patients.

	ETP (*N* = 28)	Non-ETP (*N* = 29)	*P*-value
Time of engraftment			
Absolute neutrophil count, days (range)	14 (11–21)	14 (10–20)	0.067
Platelet, days (range)	18 (12–27)	15.5 (12–72)	0.183
Infection			
CMV			
Yes	18 (64.3%)	18 (62.1%)	0.862
No	10 (35.7%)	11 (37.9%)	
EBV			
Yes	3 (10.7%)	2 (6.9%)	0.610
No	25 (89.3%)	27 (93.1%)	
Severe bacterial infection			
Yes	14 (50.0%)	9 (31.0%)	0.145
No	14 (50.0%)	20 (69.0%)	
aGVHD			
I–IV	10 (35.7%)	10 (34.5%)	0.922
II–IV	7 (25.0%)	7 (24.1%)	0.940
III–IV	5 (17.9%)	3 (10.3%)	0.664
5-year OS, %	54.74 ± 10.33	64.20 ± 10.30	0.786
5-year RFS, %	56.22 ± 10.11	57.17 ± 12.71	0.841
5-year CIR, %	30.14 ± 9.85	22.79 ± 8.24	0.774

ETP, early T-cell precursor; CMV, cytomegalovirus; EBV, Epstein–Barr virus; aGVHD, acute graft versus host disease; OS, overall survival; RFS, relapse-free survival; CIR, cumulative incidence of relapse.

## Discussion

ETP-ALL/LBL was first recognized in 2009 and defined by World Health Organization classification 2016 version as a distinct subtype of ALL due to its unique immunophenotypic and genomic profile (2, 3). Since then, many clinicians and researchers started to pay attention to the subtype. ETP-ALL is characterized by early differentiation arrest and distinct genetic and transcriptional features and thought to be a high-risk subgroup of ALL. ETP-ALL tends to be resistant to chemotherapy, and novel agents such as BCL-2 inhibitors have shown a good response for this disease. Another important and potentially curative treatment is allo-HSCT (21, 22). In this study, we summarized the characteristics of 57 T-ALL patients who received allo-HSCT at our center and compared the heterogeneity between ETP and non-ETP.

The percentage of ETP-ALL/LBL was 49.12% (28/57) in the cohort, which was higher than those in some international studies (16%–32%) (5, 12, 14, 23) but comparable with Chinese data (8, 24) (47.3%–47.6%). This may be caused by ethnic differences and may be partially due to ETP-ALL/LBL patients being more inclined to undergo allo-HSCT as a high-risk subtype.

The majority of ETP-ALL/LBL patients was male, which was consistent with previous studies (7, 8, 17, 24, 25). WBC at diagnosis was reported to be lower in ETP patients than in non-ETP patients, while the platelet count was higher (8, 14, 26). In our study, there was a trend of lower WBC and higher platelet count in ETP patients compared with non-ETP patients, but it was not significantly different [26.00 × 10^9^/L (1.48–305.64) *vs*. 51.93 × 10^9^/L (2.14–461.63), *P* = 0.158; 80.0 × 10^9^/L (25.0–327.0) *vs*. 53.5 × 10^9^/L (10.0–270.0), *P* = 0.173; respectively]. The top mutated gene in the ETP group was NOTCH1 (12/26, 46.2%), followed by NRAS, JAK3, WT1, and EZH2, while in the non-ETP group, the top five mutated genes were NOTCH1, FBXW7, NRAS, DNMT3A, and PHF6. The top mutated genes were mainly related to cytokine and RAS signaling.

A study conducted by St. Jude Children’s Research Hospital demonstrated that, with standard intensive chemotherapy, the 10-year overall survival for patients with ETP-ALL was significantly lower than for the non-ETP patients (19% *vs*. 84%, *P* < 0.0001) (2). Other two studies by MD Anderson Cancer Center and Pediatric Blood Diseases Center in our hospital also indicated inferior prognosis of ETP-ALL (5, 26). However, the Group for Research on Adult Acute Lymphoblastic Leukemia—2003 and —2005 studies showed that the 5-year overall survival for patients with ETP-ALL was not inferior to that of the non-ETP-ALL group (59.6%, 95% CI: 44.2% to 72.0% *vs*. 66.5%, 95% CI: 58.7% to 73.2%; *P* = 0.33) (14). A recent study in Chinese ALL patients also suggested a similar 2-year overall survival between the ETP and non-ETP patients (40.7% ± 8.2% *vs*. 37.9% ± 7.0%, *P* > 0.05) (8). A proportion of patients in the latter two studies received allo-HSCT other than chemotherapy alone, indicating that allo-HSCT could overcome the poor prognosis of ETP patients. In this study, we focused on ALL patients undergoing allo-HSCT and found that the 5-year OS, RFS, CIR, and NRM were not significantly different between the ETP and non-ETP patients (54.74% ± 10.33% *vs*. 64.20% ± 10.30%, hazard ratio (HR): 1.125, *P* = 0.786; 56.22% ± 10.11% *vs*. 57.17% ± 12.71%, HR: 1.091, *P* = 0.841; 30.14% ± 9.85% *vs*. 22.79% ± 8.24%, HR: 1.173, *P* = 0.774; 19.52% ± 8.99% *vs*. 25.95% ± 14.44%, HR: 0.971, *P* = 0.967). The survival of ETP-ALL/LBL patients was similar with or superior to other studies. Moreover, the OS in our study was calculated from HSCT, while in most of the previous studies it was calculated from diagnosis. Thus, our survival data was better than that of the previous studies as the median interval from diagnosis to HSCT was approximately 200 days, suggesting that allo-HSCT was an effective treatment for these patients and should be considered. However, due to the retrospective origin and small sample size, future prospective, large-scaled clinical trials are needed to investigate and confirm the results.

Furthermore, MRD status was associated with the prognosis reported by many studies (7, 12, 27). In the study, we also compared the survival of MRD-positive and MRD-negative patients and found that the 5-year OS was significantly lower in the MRD-positive patients (43.08% ± 14.67% *vs*. 59.79% ± 9.04%, *P* = 0.048).

In conclusion, in the setting of allo-HSCT, ETP-ALL/LBL and non-ETP patients could achieve similar survival. Moreover, MRD-negativity before transplantation was associated with better prognosis. Allo-HSCT should be considered for ETP patients and novel treatment strategies (such BCL-2 inhibitors, *etc.*) to eliminate MRD before transplantation could further improve the efficacy.

## Data availability statement

The raw data supporting the conclusions of this article will be made available by the authors without undue reservation.

## Ethics statement

This study was approved by the Ethics Review Committee of the Institute of Hematology, Chinese Academy of Medical Science and Peking Union Medical College and was in compliance with the Declaration of Helsinki. All patients and donors provided written informed consent for this protocol. For patients younger than 18 years old in the cohort, the consent was carried out by their parents.

## Author contributions

SF conceived and designed the study. JC analyzed the data and drafted the manuscript. SF secured financing of the study. LL, RM, AP, DY, XC, JW, YH, RZ, WZ, QM, EJ, and MH contributed to the review and editing. All authors contributed to the article and approved the submitted version.
